# Impacts of Nutrients on Alkene Biodegradation Rates and Microbial Community Composition in Enriched Consortia from Natural Inocula

**DOI:** 10.1128/spectrum.00316-22

**Published:** 2023-04-05

**Authors:** Emily Byrne, Simeon Schum, Laura Schaerer, Stephen M. Techtmann

**Affiliations:** a Department of Biological Sciences, Michigan Technological University, Houghton, Michigan, USA; b Great Lakes Research Center, Houghton, Michigan, USA; State Key Laboratory of Microbial Resources, Institute of Microbiology, Chinese Academy of Sciences

**Keywords:** Alkenes, biodegradation, nutrients, microbial communities, hydrocarbons, plastic

## Abstract

There is a growing need for biological and chemical methods for upcycling plastic waste streams. Pyrolysis processes can accelerate plastic depolymerization by breaking polyethylene into smaller alkene components which may be more biodegradable than the initial polymer. While the biodegradation of alkanes has been extensively studied, the role microorganisms play in alkene breakdown is not well understood. Alkene biodegradation holds the potential to contribute to the coupling of chemical and biological processing of polyethylene plastics. In addition, nutrient levels are known to impact rates of hydrocarbon degradation. Model alkenes were used (C6, C10, C16, and C20) to follow the breakdown capability of microbial communities from three environmental inocula in three nutrient levels over the course of 5 days. Higher-nutrient cultures were anticipated to exhibit enhanced biodegradation capabilities. Alkene mineralization was assessed by measuring CO_2_ production in the culture headspace using GC-FID (gas chromatography-flame ionization detection), and alkene breakdown was directly quantified by measuring extracted residual hydrocarbons using gas chromatography-mass spectrometry (GC/MS). Here, the efficacy of enriched consortia derived from the microbial communities of three inoculum sources (farm compost, Caspian Sea sediment, and an iron-rich sediment) at alkene breakdown was investigated over the course of 5 days across three nutrient treatments. No significant differences in CO_2_ production across nutrient levels or inoculum types were found. A high extent of biodegradation was observed in all sample types, with most samples achieving 60% to 95% biodegradation of all quantified compounds. Here, our findings indicate that alkene biodegradation is a common metabolic process in diverse environments and that nutrient levels common to culture media can support the growth of alkene-biodegrading consortia, primarily from the families *Xanthamonadaceae*, *Nocardiaceae*, and *Beijerinkiaceae*.

**IMPORTANCE** Excess plastic waste poses a major environmental problem. Microorganisms can metabolize many of the breakdown products (alkenes) of plastics. While microbial degradation of plastics is typically slow, coupling chemical and biological processing of plastics has the potential to lead to novel methods for the upcycling of plastic wastes. Here, we explored how microbial consortia derived from diverse environments metabolize alkenes, which are produced by the pyrolysis of polyolefin plastics such as HDPE, and PP. We found that microbial consortia from diverse environments can rapidly metabolize alkenes of different chain lengths. We also explored how nutrients affect the rates of alkene breakdown and the microbial diversity of the consortia. Here, the findings indicate that alkene biodegradation is a common metabolism in diverse environments (farm compost, Caspian sediment, and iron-rich sediment) and that nutrient levels common to culture medium can support growth of alkene-biodegrading consortia, primarily from families *Xanthamonadaceae*, *Nocardiaceae*, and *Beijerinkiaceae*.

## INTRODUCTION

The use of polyethylene plastics in industrial applications has surged by over 600% since the 1970s (1). Globally, over 350 million tons of plastic are produced annually, and most plastics are not recycled but rather accumulate in landfills or aquatic environments (1, 2). In 2015, most manufactured plastic and plastic waste produced consisted of polyolefin plastics such as high-density polyethylene (HDPE), low-density polyethylene, and polypropylene, with HDPE being the most common (2).

HDPE in particular is a popular plastic which is sturdy and commonly used in plastic bags, packaging, piping, and water bottles. The structural complexity of polyethylene plastics makes them recalcitrant environmental pollutants (3). One strategy to overcome the slow biodegradation of plastic is to introduce a pretreatment step to break down plastic into a more simplistic substrate for microorganisms. Pyrolysis uses high temperatures (400 to 900°C) which partially decompose plastic compounds, resulting in shorter-chain hydrocarbons and monomers. Polyethylene plastics are polymers of straight-chain alkane compounds, which are common components of crude oil mixtures. Pyrolysis treatment of polyethylene results in the formation of long-chain alkene products. Given the widespread presence of oil-degrading microorganisms in the environment, the use of environmental inocula may serve as a starting consortium for the breakdown of polyethylene plastic monomers and pyrolysis-treated polyethylene. Pyrolysis and other thermochemical processes have been previously used to process different plastic feedstocks (4, 5). However, extending the application of these techniques to the production of microbially digestible intermediate compounds under various conditions is understudied. Previous work has shown that alkenes generated from pyrolysis can be biodegraded by bacterial isolates as well as microbial communities (6–8). Gaining further understanding of microbial community responses to influxes of alkene compounds in a variety of environments may indicate the feasibility of late-stage plastic biodegradation.

Oil contamination in the environment can provide a carbon source for microbial communities in a wide range of environments. Bacteria have been known to metabolize hydrocarbon compounds in both marine and freshwater aquatic systems, as well as in sediments (9–12). Over 175 bacterial genera have been classified as oil-degraders in a variety of conditions (13), and these are widespread across environments (14). This ubiquitous nature of these bacteria in the environment is likely attributed to the presence of both aerobic and anaerobic oil-utilizing metabolisms in microbial communities (15, 16). Hydrocarbons are also naturally produced by other bacteria and algae and may serve as a natural substrate for hydrocarbon-metabolizing bacteria (17, 18). In the event of an oil spill, oil-degrading microbial taxa rapidly bloom to outcompete other microbes, dominating as much as 90% of the community within 72 h (19). Hydrocarbon contamination in the environment typically consists of a mixture of a broad range of compounds, including alkanes, alkenes, and aromatic hydrocarbons of varying sizes and complexities (20). The biodegradation of alkanes is well-characterized in aquatic systems and soils regarding both the rate of biodegradation and the composition of the associated microbial community (21–25). However, the community-wide roles of microorganisms in the biodegradation of alkene hydrocarbon compounds are less understood. Biodegradation of alkene compounds in particular not only holds significance for the role of alkenes as environmental contaminants but may also be applicable to the processing of solid industrial waste.

Hydrocarbon biodegradation rates are controlled both by the microbial community composition and *in-situ* conditions such as temperature and ambient nutrients (26, 27). Under suboptimal conditions in the environment, biodegradation of hydrocarbons is still often achievable. However, developing enrichment cultures of oil-degrading microbes operating under optimal conditions in a laboratory setting may hold potential for enhanced biodegradation and application in biological recycling and upcycling of plastics. Additionally, the use of enriched microbial consortia allows for the creation of a simplified community to study the process of biodegradation and allow for targeted questions related to hydrocarbon-degrading microorganisms (28–30). The aim of this work is to demonstrate that multiple environmental inoculum sources with differing initial microbial communities are capable of alkene biodegradation in controlled laboratory conditions under three nutrient treatments. These alkene compounds are representative of high-density polyethylene pyrolysis products. Doing this may also provide more insight as to under which nutrient conditions alkene biodegradation is most efficient.

We hypothesize that biodegradation extent and rate will vary with alkene chain length, with the catabolism of longer chain-length carbon sources possibly requiring longer lag phases in cultures. We also hypothesize that significantly different microbial communities will be selected for across inoculum sources within enrichment cultures and that distinct community formation will drive differences in the alkene biodegradation response. Because *in situ* microbial community dynamics are generally responsive to shifts in temperature, nutrients, pH, and other environmental factors, we anticipate that altering nutrient concentrations under controlled laboratory conditions will significantly influence microbial community composition and alkene breakdown rates in our cultures.

## RESULTS

### Biodegradation: CO_2_ production.

**FIG 1:**
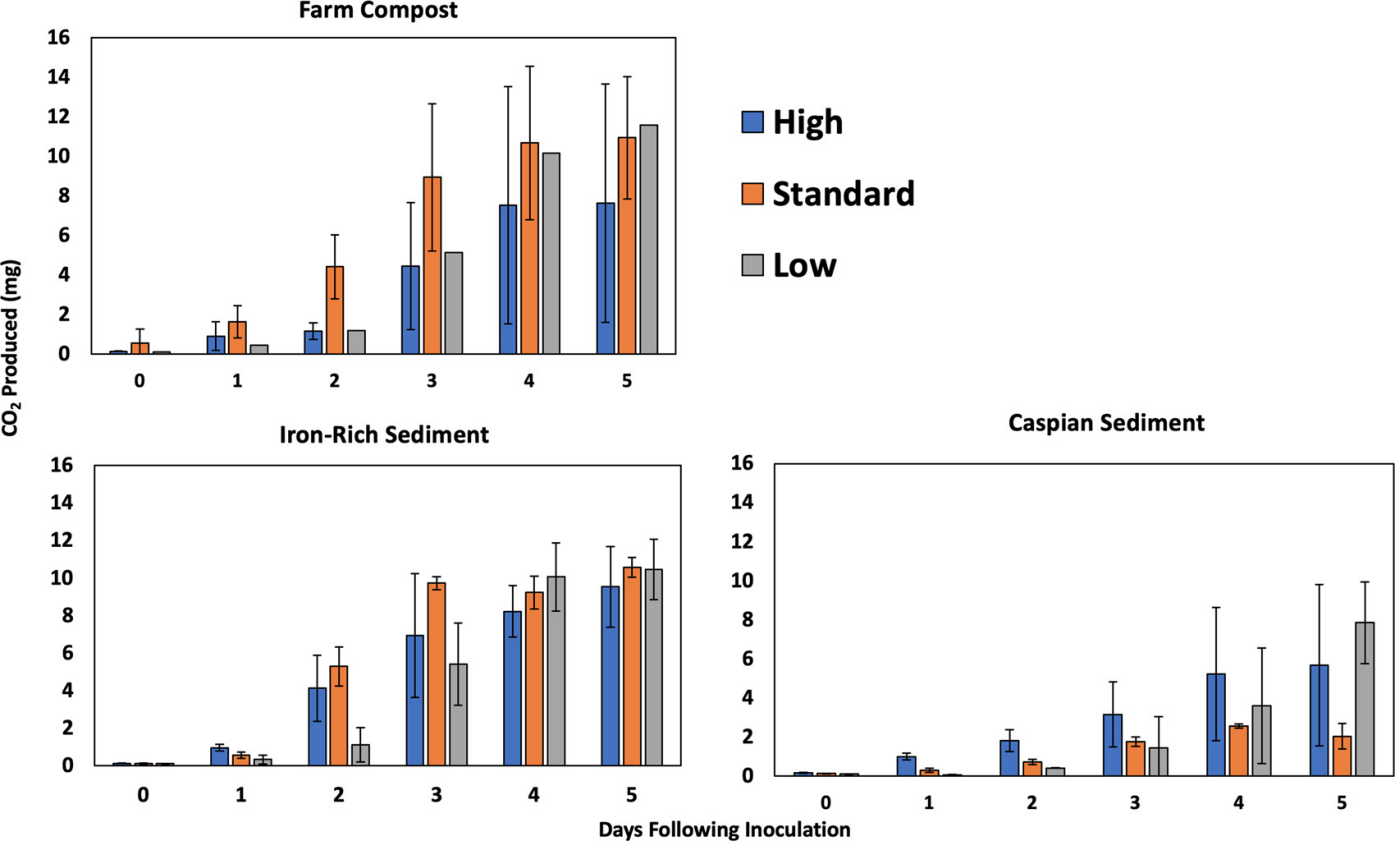
Total CO_2_ production over the course of 5 days across inoculum types and nutrient levels (high, standard, and low) in laboratory cultures indicating metabolic response to model alkene compounds. Alkene cultures were amended on day 0 with 0.5 mL of a 1:1:1:1 mix of 1-hexene, 1-decene, 1-hexadecene, and 1-eicosene. Error bars indicate standard deviation.

Here, CO_2_ production was used as an indirect means of quantifying alkene biodegradation. For each sample, CO_2_ production was calculated over the course of 5 days following inoculation (Fig. 1). The difference in total CO_2_ across treatments was not significant by Kruskal-Wallis test (*P* = 0.1712). To test our hypothesis that biodegradation rates would differ significantly between different environmental inoculum sources or nutrient levels, we compared the linear regression slopes between each nutrient-level treatment type and inoculum type combined (Table 1). A Kruskal-Wallis test revealed a *P* value of 0.4335, indicating that no statistically significant differences between the slopes of CO_2_ production in the log phase existed between any combination of inoculum sources or nutrient levels in our samples. To differentiate how much of the CO_2_ produced was more likely attributed to alkene biodegradation than to other background processes, we calculated CO_2_ production considering mean control production (Fig. S2).

**TABLE 1:** Slopes of linear regression equations for mg of CO_2_ produced per day during the exponential phase of microbial growth

Nutrient level	Inoculum source^*a*^		
	Farm compost	Iron-rich sediment	Caspian sediment
High	2.31 ± 2.67	2.99 ± 1.70	1.71 ± 1.43
Standard	3.17 ± 1.25	4.59 ± 0.15	0.92 ± 0.08
Low	3.31 ± 1.07	2.55 ± 1.00	1.59 ± 1.48

a Values given as slope ± standard deviation.

### Quantification of residual alkenes with gas chromatography-mass spectrometry (GC/MS).

**FIG 2:**
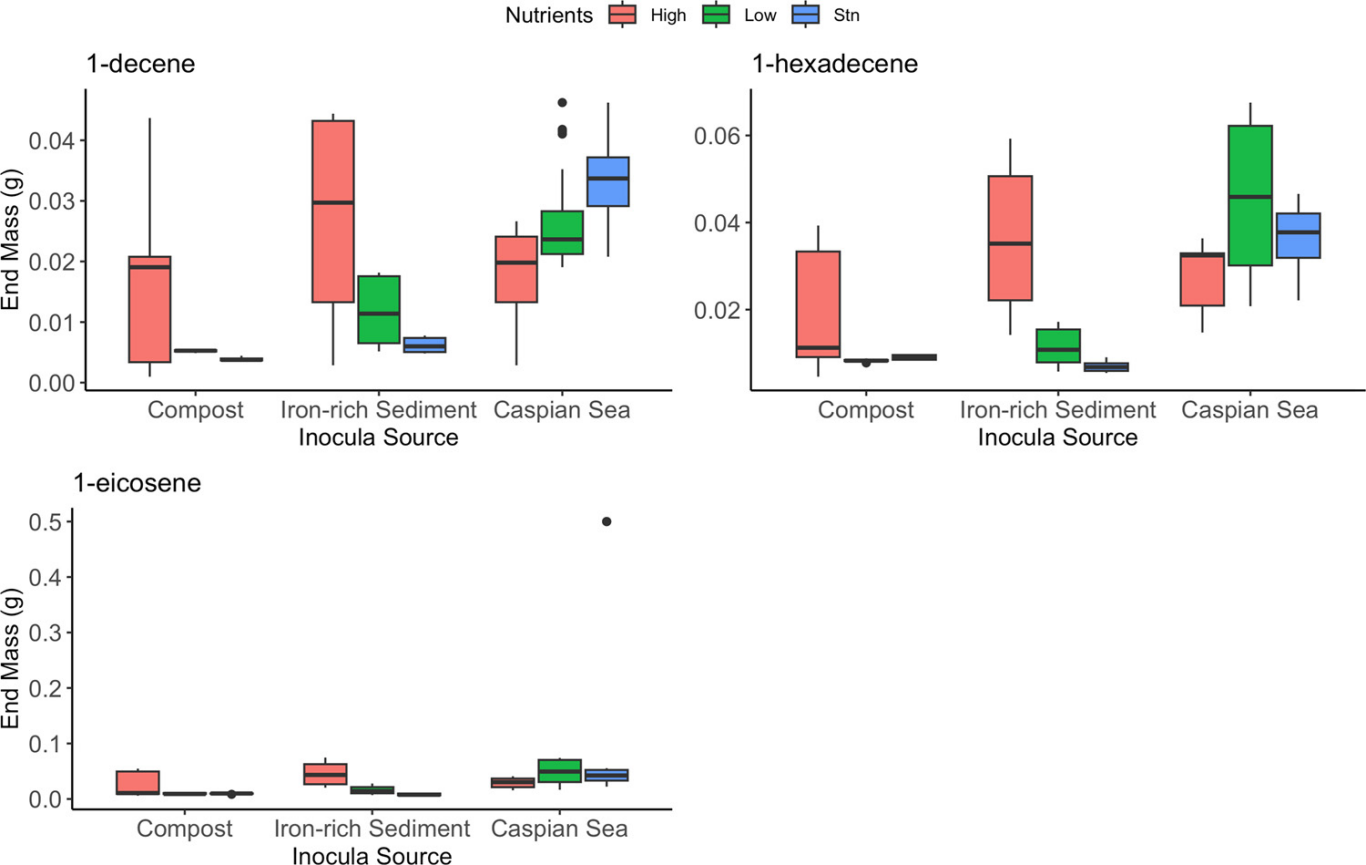
Final mass (g) of 1-decene, 1-hexadecene, and 1-eicosene in laboratory cultures consisting of various inoculum sources after the course of 5 days.

We quantified residual alkene concentrations in our cultures to determine how much of our initial alkene input was degraded over the course of 5 days (Fig. 2). We found masses (g) for 1-decene ranging from 0.04 g in the low-nutrient Caspian Sea sediment cultures to 0.0009 g in the high-nutrient compost cultures. For 1-hexadecene, masses ranged from 0.06 g in the low-nutrient Caspian Sea sediment cultures to 0.004 g in the high-nutrient compost samples. Masses of 1-eicosene ranged from 0.07 g in the low-nutrient Caspian Sea sediment samples to 0.005 g in the standard-nutrient Caspian Sea sediment samples. We were unable to quantify the hexene that was added to the cultures.

To determine whether there were significant differences in mass for each alkene compound across nutrient levels and inoculum sources, we conducted an analysis of variance (ANOVA) test, which revealed a *P* value of <0.001. To determine whether significant differences existed between specific sample types, we conducted a Tukey’s honestly significant difference (HSD) *post hoc* test. For 1-decene quantification, our results revealed significant differences between high- and low- and between high- and standard-nutrient Caspian Sea samples, as well as between high- and standard- and high- and low-nutrient level iron-rich samples (Table 2). For 1-hexadecene, significant differences existed between high- and low-nutrient Caspian Sea samples, as well as between high- and low- and between standard- and low-nutrient iron-rich sediment samples (Table 3). For 1-eicosene, no significant differences were observed between any nutrient level (Table 4).

**TABLE 2:** Tukey’s HSD *post hoc* results showing adjusted *P* values for comparisons between residual masses of 1-decene across nutrient treatments within all inoculum cultures^*a*^

	Adjusted *P*		
Sediment and nutrient level	High	Standard	Low
Caspian Sea			
High	-^*b*^	**<0.001**	**<0.001**
Standard		-	0.57
Low			-
Iron-rich			
High	-	**<0.001**	**0.01**
Standard		-	0.12
Low			-
Farm compost			
High	-	0.65	0.73
Standard		-	0.99
Low			-

a HSD, honestly significant difference. Values in bold indicate significant differences (*P* < 0.05).

b Dashes along the diagonal indicate that the samples were compared to themselves.

**TABLE 3:** Tukey’s *post hoc* results showing adjusted *P* values for comparisons between residual masses of 1-hexadecene across nutrient treatments within all inoculum cultures^*a*^

	Adjusted *P*		
Sediment and nutrient level	High	Standard	Low
Caspian Sea			
High	-^*b*^	0.41	**<0.01**
Standard		-	0.14
Low			-
Iron-rich			
High	-	0.94	**<0.01**
Standard		-	**<0.01**
Low			-
Farm compost			
High	-	0.62	0.39
Standard		-	0.99
Low			-

a Bolded values indicate significant differences (*P* < 0.05).

b Dashes along the diagonal indicate that the samples were compared to themselves.

**TABLE 4:** A summary of Tukey *post hoc* results depicting adjusted *P* values for comparisons between the residual masses of 1-eicosene across nutrient treatments within all inoculum cultures

	Adjusted *P*		
Sediment and nutrient level	High	Standard	Low
Caspian Sea			
High	-^*a*^	0.07	0.98
Standard		-	0.47
Low			-
Iron-rich			
High	-	0.47	0.77
Standard		-	0.99
Low			-
Farm compost			
High	-	0.99	0.99
Standard		-	0.99
Low			-

a Dashes along the diagonal indicate that the samples were compared to themselves.

**FIG 3:**
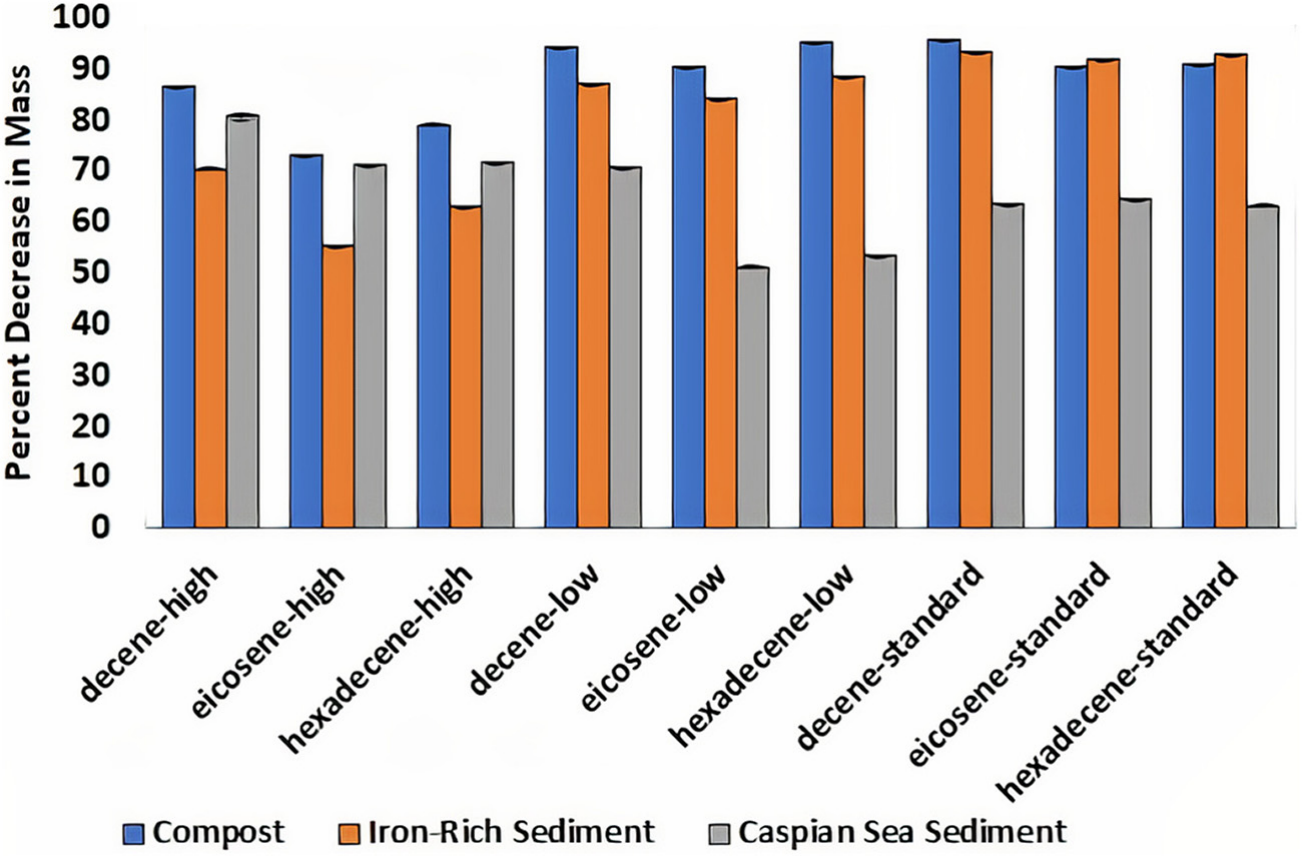
Percentage of alkenes lost during incubations. Alkene masses in different cultures were determined and converted to percentage lost based on the starting input concentrations of alkenes. The average percentage lost for the triplicate cultures for each alkene in each nutrient medium is presented here. Different inoculum sources are shown by different colors. Errors bars represent standard deviation.

We used the quantification of alkenes to determine the percent mass of degradation across all treatment types and compounds (Fig. 3). Low-nutrient compost samples displayed the highest extent of degradation for 1-hexadecene with an approximately 95% decrease in mass. Conversely, low-nutrient Caspian Sea samples displayed the lowest decrease in mass of 1-eicosene with a 49% decrease in mass.

### Protein quantification.

**FIG 4:**
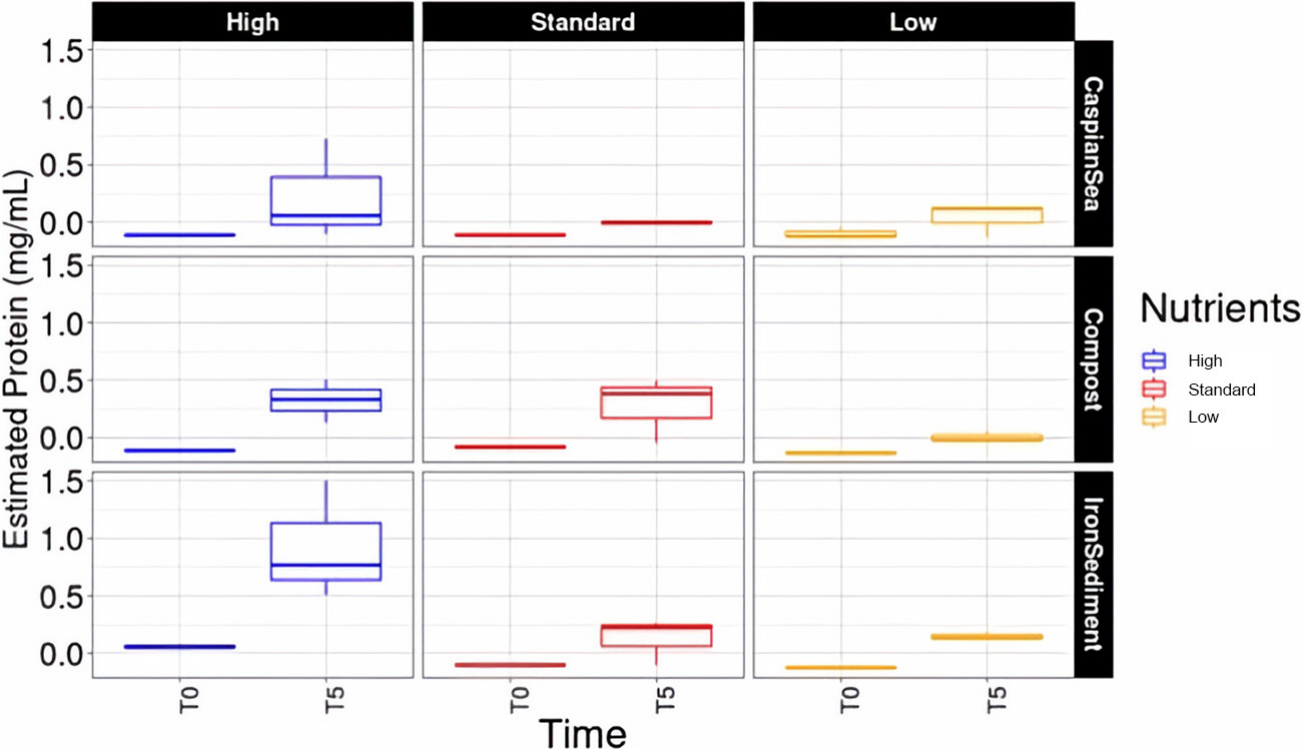
Boxplot representing protein concentrations within laboratory cultures consisting of various environmental inoculum sources after the course of 5 days. These values were obtained using a Thermo Fisher Scientific protein extraction kit and a calibration curve was created using albumin protein standards.

We quantified protein content in our cultures to estimate how much of our initial alkene input was being allocated toward assimilatory metabolism in cells. We found protein concentrations ranging from 1.49 mg/mL in high-nutrient iron-rich sediment cultures after 5 days to near 0 mg/mL in low-nutrient compost samples at the initial time point of 0 days following inoculation (Fig. 4). In all cultures, the protein content increased during the 5-day incubation, indicating that a portion of the alkenes was being assimilated into cellular biomass in the form of proteins. Proteins represent only a portion of overall cell biomass. Therefore, while protein measurements allow for the analysis of trends, they are most likely an underestimate of the biomass produced in these cultures.

### Mass balance.

To follow the flow of carbon from alkenes through this system, we attempted a rough mass balance based on the data that were measured (Table 5). The alkene input amount was based on the mass of alkenes added to each flask. We found unaccounted-for mass ranging from 0.195 ± 0.06 g in high-nutrient iron-rich sediment cultures after 5 days to 0.321 ± 0.01 g in low-nutrient compost samples (Table 5). This indicates that a large portion of the carbon is unaccounted for in our current estimates. These differences could be explained by the fact that proteins are an underestimate of total biomass; some of the alkenes may have been lost due to abiotic processes such as evaporation that were not accounted for in this mass balance.

**TABLE 5:** Mass balances for enrichment cultures from several inoculum sources grown on liquid alkene products^*a*^

Inoculum group and nutrient level	Initial alkene input (g)	Mass (g)^*b*^			
		Attributed to protein production	Attributed to CO_2_ production	Leftover of alkenes combined (GC/MS)	Unaccounted for
Farm compost					
High	0.374	0.03 ± 0.019	0.007 ± 0.006	0.06 ± 0.012	0.277 ± 0.03
Standard	0.374	0.02 ± 0.028	0.01 ± 0.003	0.03 ± 0.009	0.314 ± 0.04
Low	0.374	0.0003 ± 0.005	0.01 ± 0.001	0.04 ± 0.007	0.321 ± 0.01
Iron-rich sediment					
High	0.374	0.09 ± 0.051	0.009 ± 0.002	0.08 ± 0.016	0.195 ± 0.06
Standard	0.374	0.02 ± –0.003	0.01 ± 0.0005	0.03 ± 0.004	0.314 ± 0.007
Low	0.374	0.01 ± 0.003	0.01 ± 0.001	0.05 ± 0.008	0.304 ± 0.012
Caspian Sea sediment					
High	0.374	0.02 ± 0.040	0.005 ± 0.004	0.13 ± 0.022	0.219 ± 0.09
Standard	0.374	0.0004 ± 0.0006	0.002 ± 0.0006	0.13 ± 0.021	0.238 ± 0.02
Low	0.374	0.004 ± 0.015	0.007 ± 0.002	0.106 ± 0.054	0.257 ± 0.07

a Estimated protein content, headspace CO_2_ production, and remaining residual alkenes (C10, C16, and C20) are considered. Calculations do not account for total biomass production, for which protein production is approximately a 50% underestimation.

b Values given as means ± standard deviation.

### Microbial diversity in raw inoculum sources.

**FIG 5:**
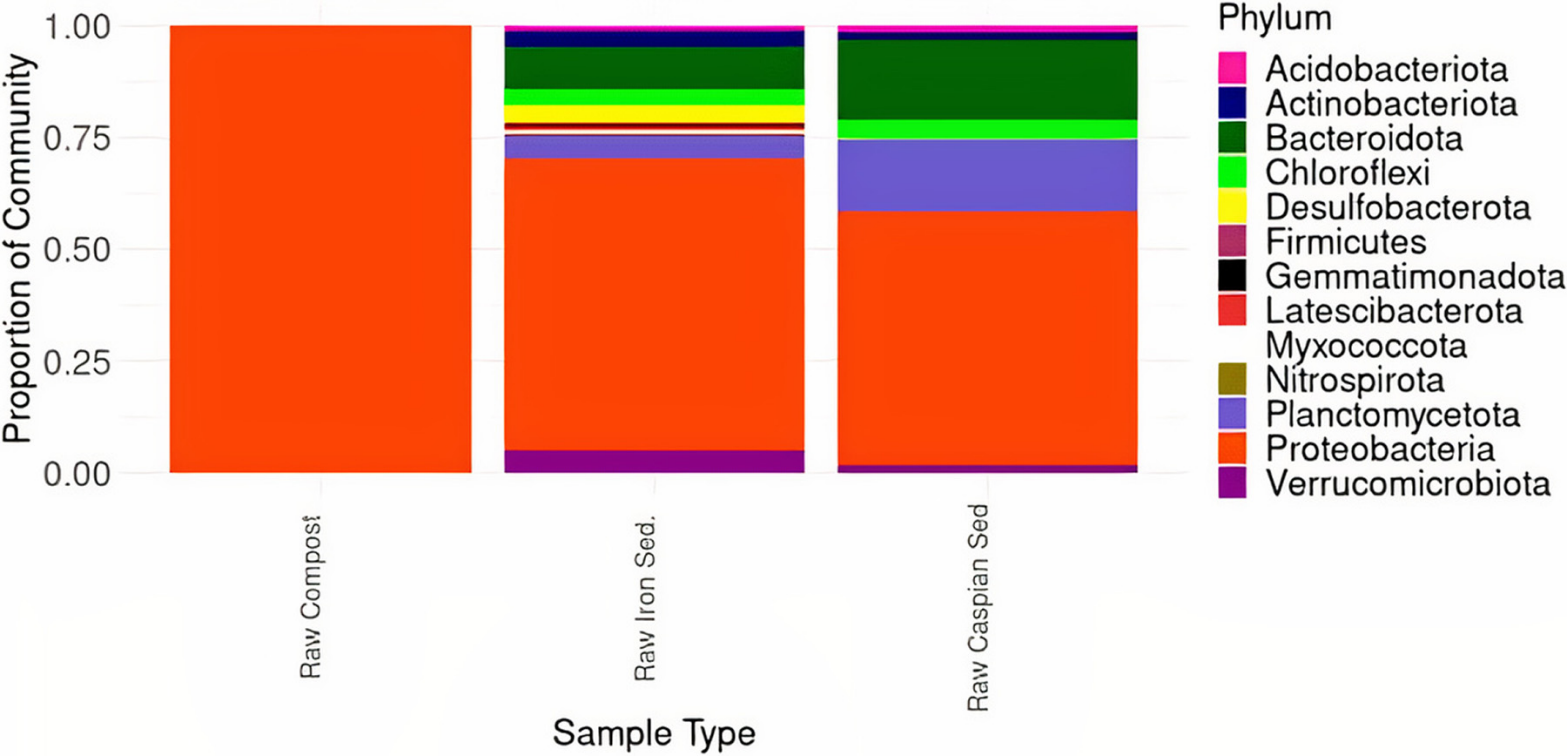
Phylum-level taxon plot depicting microbial community composition in raw inoculum samples not amended with alkene compounds. Taxonomic assignments were based on sequencing of the V4–V5 16S rRNA gene. Across all samples, 13 phyla were observed. Trends of high abundances of Proteobacteria across all inoculum types were observed, as well as increased *Bacteroidetes* in iron-rich sediment samples and Caspian Sea sediment samples.

In the 16S rRNA sequences for raw inocula without alkene amendment, we detected 26,069 amplicon sequence variants (ASVs) across 13 bacterial phyla (Fig. 5). The most prevalent phylum across the microbial communities in all inocula sources was *Proteobacteria*, with the highest number of taxa observed in iron-rich sediment inocula. The second most prevalent bacterial phylum observed was *Bacteroidetes*, from which taxa were observed in iron-rich sediment and Caspian Sea sediment. Compost samples contained less microbial diversity, containing bacteria only represented by *Proteobacteria*.

### Microbial diversity of alkene-amended cultures: alpha diversity.

**FIG 6:**
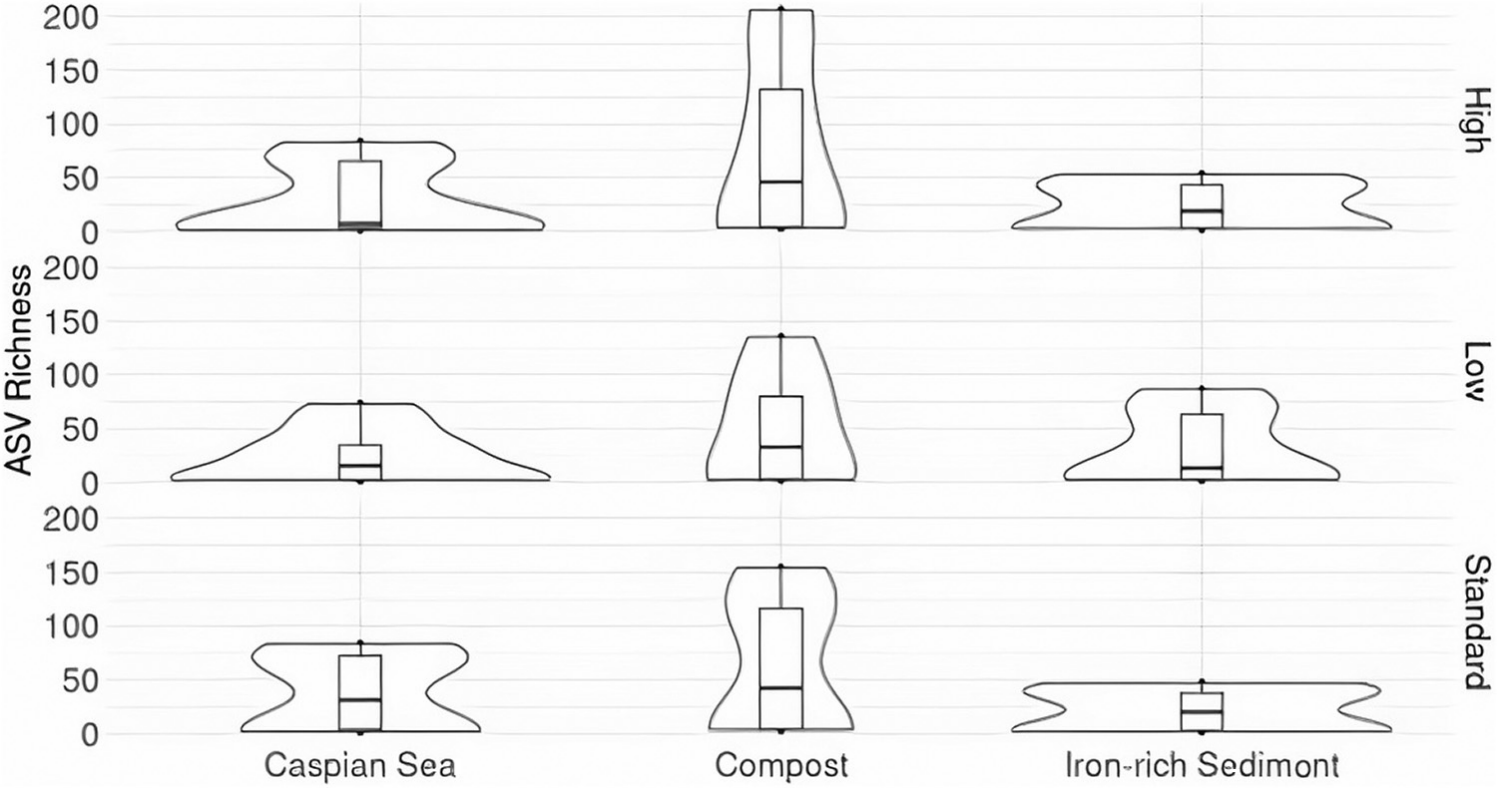
Alpha diversity represented as amplicon sequence variant (ASV) richness across Caspian Sea sediment, farm compost, and iron-rich sediment. Colors represent differences in nutrient treatment (high, standard, low). The highest diversity was present in compost samples, with the high-nutrient treatment group presenting the highest observed ASV richness. Conversely, the standard-nutrient iron-rich sediment group contained the lowest observed ASV richness.

To assess differences in community diversity between alkene-enriched microbial communities, we compared ASV richness values between compost, Caspian sediment, and iron-rich sediment samples. Alkene-amended cultures with compost inocula had the highest alpha diversity overall, and standard- and high-nutrient Caspian sediment cultures had the lowest diversity across all seasons and treatments (Fig. 6).

**FIG 7:**
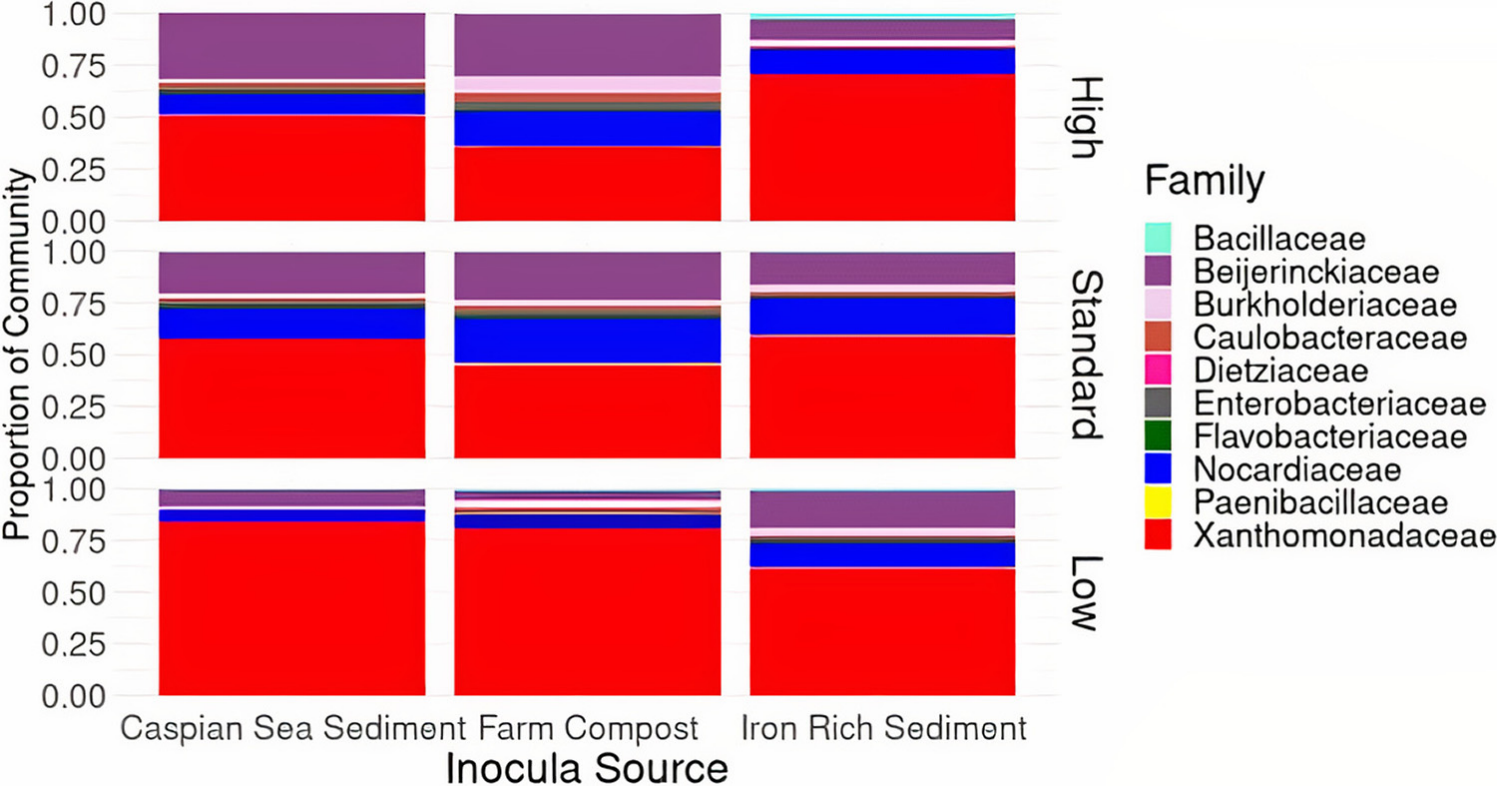
Taxon plot depicting microbial diversity across inoculum sources and nutrient levels for cultures amended with four alkene compounds (C6, C10, C16, and C20) after the course of 5 days.

We visualized the spread of bacterial classes in our samples using a taxon plot to assess microbial diversity across all inoculum sources and nutrient levels. The consortia contained members from four phyla (*Proteobacteria*, *Firmicutes*, *Actinobacteria*, and *Bacteriodetes*) and nine total bacterial families across all sample types. *Xanthomonadaceae* were highly abundant across all sample types, with *Xanthomonadaceae* being most abundant in low-nutrient compost samples at approximately 90% of the total community composition (Fig. 7). Standard nutrient samples across all inoculum types contained increased *Nocardiaceae* (15% to 20%) relative to high- and low-nutrient samples.

**FIG 8:**
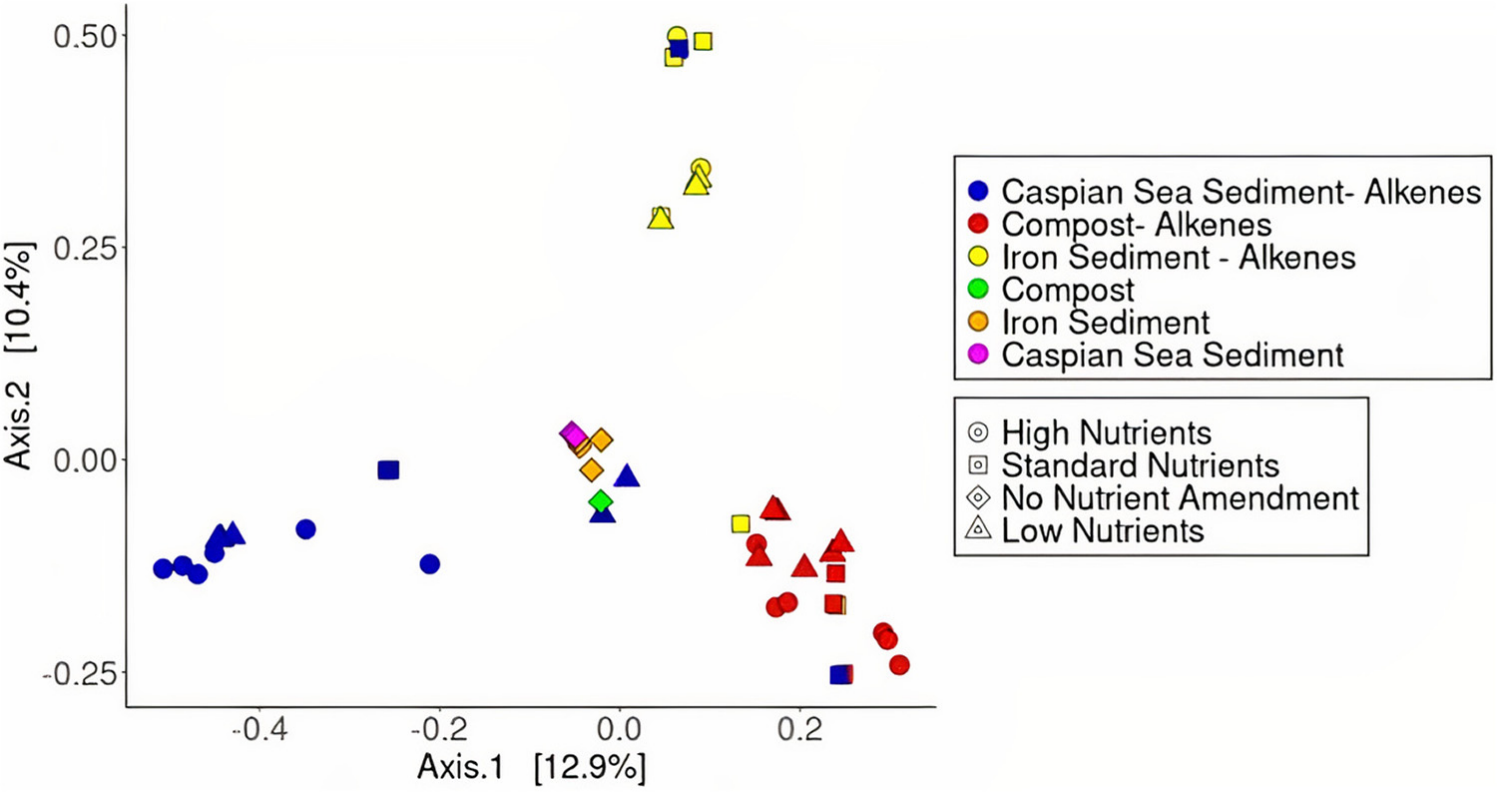
A principal coordinate analysis plot showing diversity between samples for bacterial 16S rRNA sequences across three nutrient levels and inoculum types amended with a mixture of alkene compounds (C6, C10, C16, and C20). Closer clustering of samples indicates increased similarity in ASV composition.

We conducted a principal coordinate analysis (PCoA) to visualize shifts in microbial community composition across treatments with the Bray-Curtis dissimilarity matrix. We observed distinct clustering of samples based on 16S rRNA sequences within each inoculum type (Fig. 8). Raw inoculum samples with no alkene amendment exhibited clustering in the middle of the plot, indicating that the addition of alkenes caused the microbial communities to diverge (Fig. 8). Sample clustering by nutrient level was less distinct but may indicate gradual trends in microbial community composition shifts when nutrient contents are enhanced. Overall, the first two dimensions of the PCoA plot explained 23.3% of the variation. We used a permutational multivariate ANOVA (PERMANOVA) test to determine whether statistically significant microbial community composition shifts were present across treatments. PERMANOVA was also used to determine the variation explained by differences in microbial community structure between inoculum types and nutrient levels in the PCoA. We used the “adonis2” program as part of the vegan package in R to perform the PERMANOVA test to determine between which sample types significant differences were present. We observed significant differences between the microbial communities of each inoculum type when grouping all nutrient treatments together (Table 6, Table S1).

**TABLE 6:** Summary of PERMANOVA results showing R^2^ values between microbial communities across nutrient treatments within inoculum cultures^*a*^

	*R*^2^ (F-model value)		
Sediment and nutrient level	High	Standard	Low
Caspian Sea			
High	-^*b*^	**0.37573 (5.4169)**	**0.36331 (5.7063)**
Standard		-	**0.2724 (3.3694)**
Low			-
Iron-rich			
High	-	0.27787 (1.5392)	0.13407 (0.92899)
Standard		-	0.19977 (1.4978)
Low			-
Farm compost			
High	-	**0.35346 (5.4669)**	**0.3504 (3.7758)**
Standard		-	**0.37827 (4.259)**
Low			-

a PERMANOVA, permutational multivariate analysis of variance. Bold values indicate that the probability of >F for a comparison was <0.01.

b Dashes along the diagonal indicate that the samples were compared to themselves.

### Differential abundance analysis.

To begin to understand which bacteria were enriched in different treatments, we performed differential abundance analysis using DESeq2 (14). We found significantly enriched bacterial ASVs between nutrient groups in every inoculum type (Table 7). All significantly enriched ASVs belonged to one of three bacterial families: *Xanthomonadaceae*, *Beijerinkiaceae*, or *Nocardiaceae*. All the enriched ASVs from *Xanthomonadaceae* were classified as members of the genus *Stenotrophomonas*. Similarly, the significantly enriched ASVs classified as *Beijerinckiaceae* were members of the genus *Methylobacterium*. The genera *Gordonia* and *Rhodococcus* were the only taxonomic assignments represented across enriched ASVs within *Nocardiaceae*.

**TABLE 7:** Summary of significantly enriched ASVs within inoculum types across nutrient levels^*a*^

Inoculum source and nutrient level comparison^*b*^	ASVs enriched in the first group (*n*)	Bacterial taxa enriched in the first group^*c*^
Caspian Sea		
**High** vs. standard	8	*Xanthomonadaceae* (6), *Nocardiaceae* (2)
**Standard** vs. high	6	*Xanthomonadaceae* (4), *Beijerinckiaceae* (2)
**Standard** vs. low	3	*Xanthomonadaceae* (1), *Beijerinckiaceae* (2)
**Low** vs. standard	2	*Xanthomonadaceae*
**High** vs. low	3	*Xanthomonadaceae* (2), *Beijerinckiaceae* (1)
**Low** vs. high	8	*Xanthomonadaceae* (5), *Beijerinckiaceae* (1), *Nocardiaceae* (2)
Iron-rich sediment		
**Standard** vs. high	3	*Xanthomonadaceae* (1), *Beijerinckiaceae* (1), *Nocardiaceae* (1)
**High** vs. low	4	*Xanthomonadaceae*
**Low** vs. high	2	*Xanthomonadaceae* (1), *Beijerinckiaceae* (1)
Farm compost		
**High** vs. standard	2	*Xanthomonadaceae* (1), *Nocardiaceae* (1)
**Standard** vs. high	15	*Xanthomonadaceae* (6), *Beijerinckiaceae* (5), *Nocardiaceae* (4)
**Low** vs. standard	3	*Xanthomonadaceae*
**High** vs. low	3	*Xanthomonadaceae* (1), *Beijerinckiaceae* (1), *Nocardiaceae* (1)

a ASV, amplicon sequence variant.

b Bold font indicates that the ASVs were enriched in this nutrient level relative to the other.

c Parentheses show the number of ASVs from each taxonomic group that were enriched in the nutrient level in bold. All significantly enriched ASVs belonged to the families *Xanthomonadaceae*, *Beijerinkiaceae*, and *Nocardiaceae*.

## DISCUSSION

Overall, these results indicate that alkenes can be metabolized by diverse consortia derived from multiple environments across a range of nutrient conditions. No significant differences in either total CO_2_ production or CO_2_ production rates in culture headspace across all treatment types were observed after the course of 5 days. Given that CO_2_ production did not display significant differences under varying nutrient levels despite them consisting of the same starting environmental inocula (with significant differences in microbial community composition), it can be inferred that nutrient availability may not be the most critical controlling factor for alkene biodegradation rates under these culture conditions. It is possible that even our lowest nutrient condition (0.05 g nitrogen and 0.09 g phosphorous) is substantially more nutrients than what the microbial communities would likely be exposed to in the environment, and thus did not represent nutrient-limited conditions. More work is needed to fully understand the impacts of nutrients on the biodegradation of alkene compounds in environmental settings and enrichment cultures, as our experiment only accounts for the impacts of nutrient biodegradation of four model compounds (C6, C10, C16, and C20).

We observed on average between 60% and 100% biodegradation of our chosen alkenes over the course of 5 days (Fig. 3), along with significant selection pressure within the microbial community (Fig. 5). Our results align with previous work characterizing the selection of microbial communities contributing to alkane breakdown (30). One consideration not accounted for in this study is the possibility that residual alkenes remained in culture from the transfers before we began measurements, possibly impacting biodegradation results. Specialized communities were also selected for in our consortia for alkene breakdown. Longer carbon chains and increasing complex chemical structures, such as branching, likely play a role in the alkene biodegradation rates of recalcitrant pollutants. Previous work has suggested that biodegradation is fastest for alkanes, followed by alkenes, then branched compounds, and then aromatics (31–33). Gaining further insight into the roles other environmental factors, such as temperature, play in biodegradation would be beneficial for scaling up microbial-aided alkene biodegradation.

Another consideration for our study results is the partitioning of the alkene carbon sources into dissimilatory and assimilatory metabolism sources. Regarding protein production in cultures, low-nutrient samples across all inoculum sources exhibited distinctly lower protein concentrations relative to high-nutrient samples. However, when observing CO_2_ production over the same length of time, we did not observe significant differences between high- and low-nutrient samples. This suggests that the metrics of dissimilatory metabolism, such as CO_2_ production, may not be as heavily impacted as cell growth metrics by nutrient content in cultures. Furthermore, more than one metabolism-monitoring technique may be required to discern which treatment conditions are most efficient for optimizing alkene breakdown. Bacterial growth efficiency is defined as the quantity of biomass synthesized per unit of substrate assimilated (34). In natural settings, this can be measured as the ratio of bacterial production to bacterial respiration. Since bacterial production is measured as biomass production and respiration can be measured as CO_2_ production, it is possible to consider these results from the perspective of bacterial growth efficiency. Previous studies have shown that an increased supply of nutrients results in increased bacterial growth efficiency (35, 36). High-nutrient enrichments showed a trend of increased protein production after the course of 5 days relative to low-nutrient enrichments. Our results also show that low-nutrient enrichments displayed trends of increased total CO_2_ produced relative to high-nutrient enrichments (Fig. 1). This may indicate that high-nutrient enrichments possess higher growth efficiency ratios than low-nutrient enrichments.

In general, microbial diversity in the environment tends to be higher than microbial diversity in these laboratory consortia. This is partially because a large percentage of environmental microbes resist culturing (35). The selection for an alkene-degrading consortia resulted in decreased bacterial diversity relative to the raw inocula (Fig. 5), indicating that the addition of alkenes and the culture conditions used present strong selection pressure for certain taxa in these cultures. Also, other factors such as nutrient limitation and environmental constraints, such as temperature, may control the extent of bacterial selection in response to alkenes in the environment. Here, CO_2_ production measurements indicate that the enriched cultures had reached stationary phase by the end of the 5-day experiment.

Furthermore, we were able to enrich taxa from similar bacterial families even though we used different nutrient treatments and started with inocula with distinct community compositions. This suggests that alkene-degrading taxa in environmental systems are ubiquitous in many locations. Bacteria from three families were identified as being significantly enriched across all nutrient levels and inoculum types: *Xanthomonadaceae*, *Beijerinckiaceae*, and *Nocardiaceae*. ASVs within *Xanthomonadaceae* were significantly enriched in every inoculum and nutrient level except for low-nutrient iron-rich sediment samples. ASVs within *Beijerinckiaceae* was significantly enriched in standard- and low-nutrient Caspian samples, in all nutrient levels of iron-rich sediment samples, and in standard-nutrient samples from compost inocula. ASVs from *Nocardiaceae* were enriched in high-nutrient Caspian samples, standard-nutrient iron-rich samples, and high- and standard-nutrient compost samples. All three enriched bacterial families have been previously observed to play roles in hydrocarbon biodegradation (37–39). Microbes from *Xanthomonadaceae* in particular were previously observed to be enriched in ethylbenzene-enriched soil microcosms (40). The widespread presence of enriched ASVs from each of these families within various inocula types may indicate that the bloom of bacteria after exposure to alkenes may not directly be impacted by the starting microbial community. Additionally, the lack of a significant presence of *Nocardiaceae* in low-nutrient samples may indicate that other bacterial families are more strongly selected for and outcompete them under lower nutrient conditions.

In our experiment, we only examined bacterial diversity. It is possible that fungi are present in our cultures and contribute to alkene biodegradation across nutrient levels. Previous work indicates that fungi are important key players for the biodegradation of other recalcitrant compounds such as lignin (41), and therefore may be important to consider in the context of our environmental consortium. Future work that focuses on sequencing of universal regions of fungal genomes such as internal transcribed spacer regions would provide more insight into the fungal community dynamics contributing to alkene breakdown.

We hypothesized that significantly different microbial communities would be selected for across inoculum sources within enrichment cultures and that distinctive community formation would drive differences in alkene biodegradation responses. In our cultures, we observed convergence of microbial communities across treatment types and inoculum sources, despite beginning with three distinct microbial communities from different environments. Only three bacterial families across two phyla were found to have significantly enriched taxa despite the 13 bacterial phyla represented in the initial community sequences from raw inocula. Sample types diverged at the genus level, which likely caused the divergence observed in the PCoA. Although it is contradictory to our initial hypothesis, the selection of a specialized microbial community may suggest that cultures from various environmental inocula are adaptable to alkene sources of varying chain lengths, such as those in this study.

### Conclusions.

Our goal was to quantify alkene breakdown in laboratory cultures and monitor changes in microbial communities to draw associations as to which microorganisms may play a significant role in alkene breakdown. We found no significant differences in CO_2_ production across nutrient levels or inoculum types. We observed a high extent of biodegradation in all sample types, with the majority of samples achieving 60% to 95% biodegradation of all quantified compounds. Microbial community diversity analyses indicated that cultures derived from environmental inocula from various starting microbial assemblages converged to display significant overlap of bacterial families. Significantly enriched families across all inocula types included ASVs from the families *Xanthomonadaceae*, *Nocardiaceae*, and *Beijerinckiaceae*. These results ultimately suggest that the microorganisms necessary to achieve alkene breakdown may be present in a variety of environmental microbial assemblages and are strongly selected for under optimized conditions such as nutrient amendment.

## MATERIALS AND METHODS

### Sample collection.

To demonstrate the efficiency of alkene biodegradation in a variety of microbial communities, three different inocula sources were chosen. Various Caspian Sea sediment cores from the southern basin were sampled at 39.7455, 50.4806 degrees latitude and longitude (42). Iron-rich sediment was collected from the Spurr River in Michigamme, Michigan (46.532, −88.141). Vermicompost samples were collected from a farm in Calumet, Michigan (47.211, −88.553). Environmental inoculum samples were collected in sterile containers and stored at 4°C until use in cultures. These samples represent a range of settings from terrestrial systems, freshwater aquatic, and marine locations.

### Enrichment cultures.

Cultures under each inoculum type and nutrient treatment were assembled in triplicate. Bushnell Haas medium was used in this study due to its lack of carbon sources [0.2 g MgSO_4_, 0.02 g CaCl_2_, 1 g KH_2_PO_4_, 1 g (NH_4_)_3_PO_4_, 1 g KNO_3_, 0.05 g FeCl_3_]. In each replicate, 1 g of environmental inoculum was combined with 100 mL of either low-nutrient (0.25× concentrated), standard, or high-nutrient (2× concentrated) Bushnell-Haas medium. Nitrogen and phosphorous levels present in each treatment condition as ammonium phosphate and potassium nitrate were as follows: 0.46 g/L N and 0.77 g/L P (high), 0.23 g/L N and 0.38 g/L P (standard), and 0.05 g/L N and 0.09 g/L P (low). As a carbon source, 0.5 mL of a 1:1:1:1 mixture of 1-hexene, 1-decene, 1-hexadecene, and and-1-eicosene was added. These were chosen to represent a range of carbon lengths of olefin compounds. Cultures were inoculated in 250 mL flasks and placed on a stir plate with Teflon coated stir bars at 200 rotations per minute at room temperature (23°C). After 1 week, 7 mL of each culture was transferred to a new flask with 93 mL of fresh Bushnell Haas medium and 0.5 mL of the same alkene mixture. Cultures were transferred twice before a final transfer to begin growth measurements. In the final transfer, 7 mL was transferred into fresh medium with the appropriate nutrient concentration and 0.5 mL of alkene mixture. A control set of flasks was also assembled with uninoculated Bushnell Haas medium. A graphic depicting a more detailed representation of culture setup and subsampling protocol can be accessed in Fig. S1.

### Biodegradation rate determination.

To assess culture response to alkenes, we measured carbon dioxide production rates as a proxy for alkene biodegradation. Each day for 5 days, a 1-mL sample from the headspace of each flask was analyzed with GC-FID (gas chromatography-flame ionization detection) with a methanizer using the HayeSep D column. After sampling, each flask was opened daily for 1 min to allow gas exchange and calculation of daily CO_2_ production. We did not account for CO_2_ reentering the flasks during this period in our calculations of respiration rates. One consideration for future experiments could be using longer timelines for flushing the headspace of cultures, as in this experiment we flushed cultures for 1 min. We used a linear calibration line of best-fit relating GC-FID CO_2_ peak area values to estimated gaseous volume with known volumes of a standard mixture of gases with 1% CO_2_, 1% CO, 1% H_2_, 1% CH_4_, and 1% O_2_ balanced with nitrogen. CO_2_ volume values were converted to mass (mg) for determination of CO_2_ production using the ideal gas law equation and scaled to represent the entire headspace of each flask.

Because these cultures were grown using batch culture, we observed typical growth phases. To determine the rates of CO_2_ production, we calculated linear regression lines for CO_2_ produced between time points representative of the log phases of microbial growth determined using OD_600_ (optical density at 600 nm) measurements. The slope of these regression lines was used as the rate constant for CO_2_ produced during exponential CO_2_ production. To test our hypothesis that microbial communities from different inoculum sources and nutrient levels would result in varying biodegradation rates, the slopes of the regression lines for each treatment were compared using a nonparametric Kruskal-Wallis test because sample data did not meet the assumptions for an ANOVA. To identify between which sample types a significant difference occurred, the “Dunn” package in R was used to perform a *post hoc* test.

### Quantifying residual alkene compounds.

After 5 days, 70 mL of each 100-mL culture was subsampled and frozen in amber glass bottles until hydrocarbon extraction. For extractions, 70 mL of dichloromethane (DCM) was used to rinse residual hydrocarbon compounds in the bottles and then added as a solvent to each sample in separatory funnels. Funnels were shaken vigorously for 1 min and left to settle into nonpolar and polar fractions for approximately 1 h. The miscible fraction containing nonpolar compounds was poured into amber glass vials through a 47-mm Watman glass fiber filter for each sample to remove residual particles and biomass that would interfere with GC/MS analysis. Filters were rinsed with 5 mL of DCM to remove any residual alkene compounds. The solvent fraction was blown down with nitrogen gas to a volume of <2 mL. All extractions were stored at 4°C until GC/MS analysis.

### Quantification of alkenes with GC/MS.

The volume of all extracts was measured for subsequent quantification of alkene mass. Hydrocarbon extracts were diluted to 1% of their initial concentration in DCM. Next, 10 μL of 1,000 μg/mL 1,3,5-trichlorobenzene (SPEX Certiprep) was added to each diluted sample as an internal standard to correct for injection error by the autosampler. Samples were analyzed using a coupled Trace 1310 gas chromatograph and ITQ 1100 ion trap mass spectrometer (Thermo Fisher Scientific) equipped with a TriPlus RSH autosampler (Thermo Fisher Scientific). The GC was heated at 65°C, held for 3 min, then heated from 65°C to 240°C, ramping at 30°C until reaching a temperature of 240°C, and held for 1 min using a TG-5MS column (Thermo Fisher Scientific). The mass spectrometer ion source temperature was heated to 275°C. A full scan was conducted with an *m/z* ratio of 20 to 300, with a max ion time of 75 μs. An autosampler was used for injections and was rinsed three times with 3 μL of DCM between each run. Injection volumes of 1 μL per sample were used for analysis. Each sample was run in a series of five technical replicates. Calibration curves were developed for 1-decene, 1-hexadecene, and 1-eicosene using the ratio of their peak area to the peak area of the internal standard (10 μL of 1,000 μg/L 1,3,5-trichlorobenzene [SPEX Certiprep]). These ratios were used to generate calibration curves to quantify the amount of each compound in the diluted samples. The concentrations of these diluted samples were then back-calculated to the concentration of the compounds in the original non-diluted hydrocarbon extracts.

To determine whether there were statistical differences in alkene concentrations between sample types and nutrient sources, we started by using the “pwr” package in R (43) to determine whether the sample sizes of each inoculum source and nutrient treatment met the assumptions for statistical comparison with ANOVA. We found that with the number of technical replicates, we had sufficient statistical power to do so with an alpha value of 0.05 and a power value of 0.8. Hence, we utilized the ANOVA test in base R to assess statistical differences in alkene concentrations among sample types. Pairwise comparisons between sample types were done using the *post hoc* Tukey’s HSD function.

### Culture growth and protein quantification.

To quantify growth partitioned to biomass production in our cultures, we quantified protein content in the culture at the initial and final time points. A detergent solution was made using 1 g of sodium dodecyl sulfate, 5 mL Tris (pH = 8), and 9.5 mL sterilized water. Next, 75 μL of the detergent solution was combined with 75 μL of culture subsample from each time point, for a total volume of 150 μL, in a 96-well plate and heated in a thermocycler at 100°C for 10 min. The Pierce BCA (bicinchoninic acid assay) Protein Assay kit produced by Thermo Fisher Scientific was used. Then, 200 μL of the BCA working reagents was mixed with 10 μL of boiled sample in a separate 96-well plate. The plate was incubated at 37°C for 30 min before absorbance values were measured using a spectrophotometer at 562 nm for each well. A standard curve was created with various concentrations of an Albumin protein stock standard solution to correlate absorbance with protein concentration.

### 16S rRNA sequencing.

From each culture, 30 mL was vacuum-filtered on 0.03-μm Sterlitech polyethersulfone filters and stored at −80°C. Sample DNA was extracted from the entire filter using a ZymoBIOMICs DNA Microprep kit (Zymo Research Corporation, Irvine, CA) following the manufacturer’s specifications with the exception of homogenizing samples for 200 s at 5.5 m/s using a FastPrep 5G (MP Biomedicals). DNA was also extracted from 1 g of each raw inoculum source to represent the starting microbial community before alkene amendment. Extracted DNA was then prepared for high-throughput sequencing to construction V4–V5 16S rRNA gene libraries using a modified version of the Illumina 16S rRNA library preparation protocol. Briefly, the V4–V5 hypervariable regions of the 16S rRNA gene were amplified using the 515F-Y (5′-GTGYCAGCMGCCGCGGTAA) and 926R (5′-CCGYCAATTYMTTTRAGTTT) primers (44) with 25 cycles. After PCR purification using Axyprep magnetic beads (Corning), sequencing indexing barcodes were added through a second 8-cycle PCR step. To account for possible contamination of our reagents at each step, we ran blank wells containing no added DNA. The barcoded amplicons were purified using an Axyprep Mag PCR Clean-Up kit. A PicoGreen dsDNA quantification assay (Thermo Fisher Scientific) was used to determine the concentrations of each library pool. The libraries were then pooled to produce a single 4-nM pool for sequencing. This pool was sequenced using a v3 600-cycle Illumina kit using Michigan Technological University’s MiSeq instrument. Raw sequencing data have been deposited in the SRA and are associated with BioProject ID PRJNA733145.

### 16S rRNA read analysis.

All sequencing data were analyzed in R (30).We used the package DADA2 (divisive amplicon denoising algorithm) to process and trim sequencing reads (43). Reads falling below a quality score of 30 were trimmed. The forward and reverse reads were trimmed at 250 cycles. Reads were denoised, forward and reverse reads were merged, and chimeras were identified. For the amplicon sequence variant table, samples were rarified to a depth of 1,000 reads. A taxon table was constructed with the package “phyloseq” using taxonomical assignments from the SILVA v132 data set for DADA2 (45). Sequences from mitochondria and chloroplasts were filtered out of the data set. The “estimate_richness” function in phyloseq was used to determine alpha diversity on the non-rarified taxon table.

All statistical analyses were carried out using R (30). We used the “pwr” package in R to determine whether sample size between each inoculum source and nutrient level met the assumptions for statistical comparison with ANOVA and found that we did not have sufficient statistical power to do so with a power value of 0.8 and a significance level of 0.05 (46). As a result, we utilized a nonparametric Kruskal-Wallis test to assess statistical differences in microbial communities among inoculum types and nutrient levels. Pairwise comparisons between sample types were done using the Dunn function in the package “FSA” (47). PCoA was completed using the “phyloseq” package to visualize microbial community shifts across treatments with the Bray-Curtis dissimilarity matrix. A PERMANOVA test was used to determine the extent of variation explained by the differences in microbial community structure between treatment types in the PCoA. PERMANOVA as implemented in the “Adonis2” function was used to determine the percentage of variation that could be explained by the PCoA within the “vegan” package (48).

### Differential abundance analysis.

To determine whether significantly enriched ASVs were present between inoculum sources and nutrient levels in the 16S rRNA reads, we used the package “DESeq2” (17) to conduct differential abundance analyses between sample variables with an alpha of 0.01. ASVs with a log_2_-fold value of >2 or <-2 were considered to represent significant differences in abundance.

### Data availability.

Raw sequencing data have been deposited in the SRA and are associated with BioProject ID PRJNA733145.
