# Supplementary material for: Impacts of Nutrients on Alkene Biodegradation Rates and Microbial Community Composition in Enriched Consortia from Natural Inocula
Source: Microbiol Spectr. 2023 Apr 5;11(3):e00316-22. doi: 10.1128/spectrum.00316-22 (PMC10269803; doi:10.1128/spectrum.00316-22)
Supplement: Supplemental file 1 — Supplemental material. Download spectrum.00316-22-s0001.pdf, PDF file, 0.3 MB [file spectrum.00316-22-s0001.pdf]

Impacts of Nutrients on Alkene Biodegradation Rates and Microbial Community Composition in Enriched Consortia from Natural Inocula

Emily Byrne[a], Simeon Schum[b], Laura Schaerer[a], Stephen M Techtmann[a]*

[a]Department of Biological Sciences, Michigan Technological University, 1400 Townsend Drive, Houghton, MI, USA

[b]Great Lakes Research Center, 100 Phoenix Drive, Houghton, MI, USA

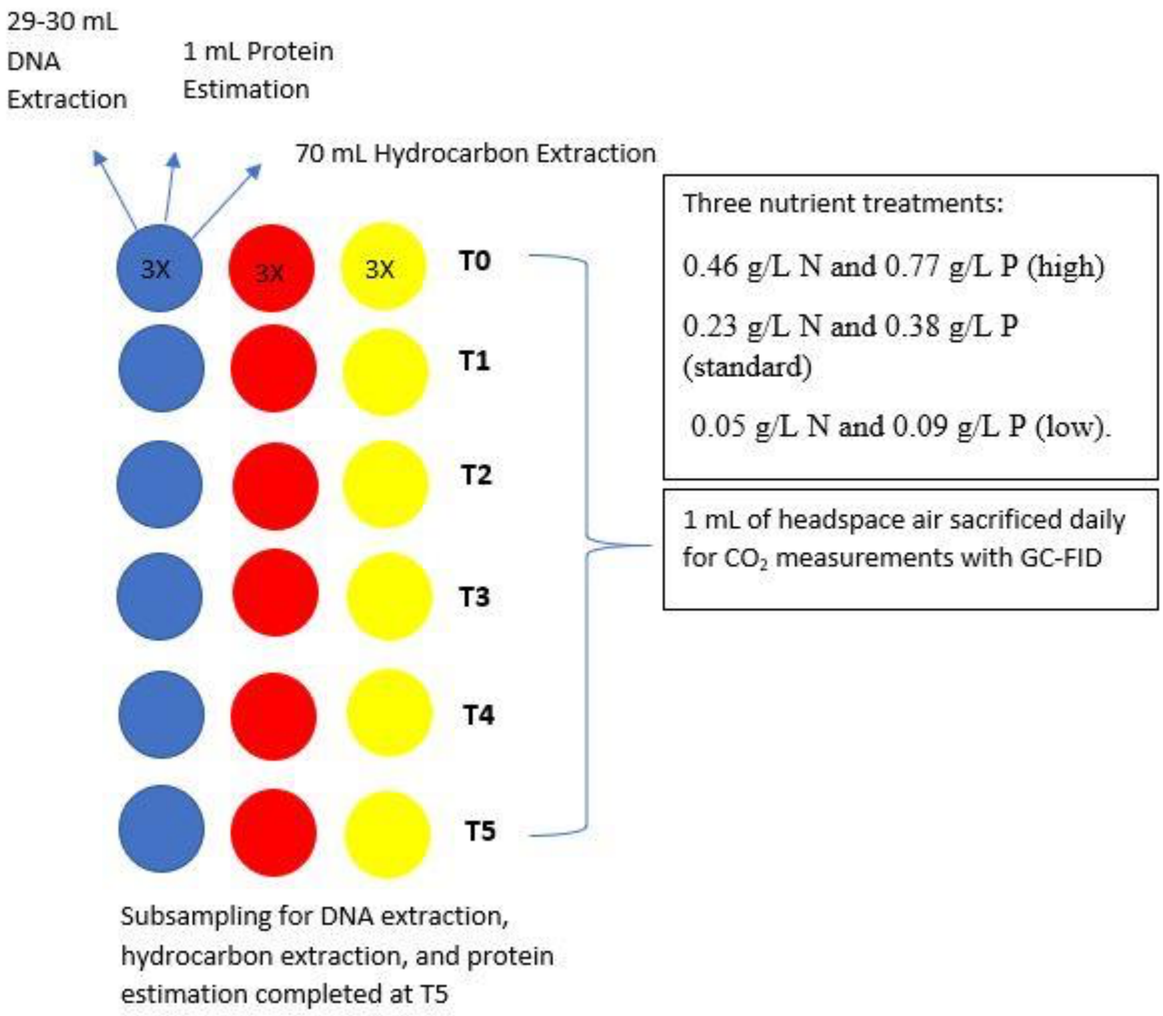

Figure S1. A more detailed graphic depicting our experimental design for 16S rRNA sequencing and subsampling for CO_2 measurements, hydrocarbon extractions, and protein estimation.

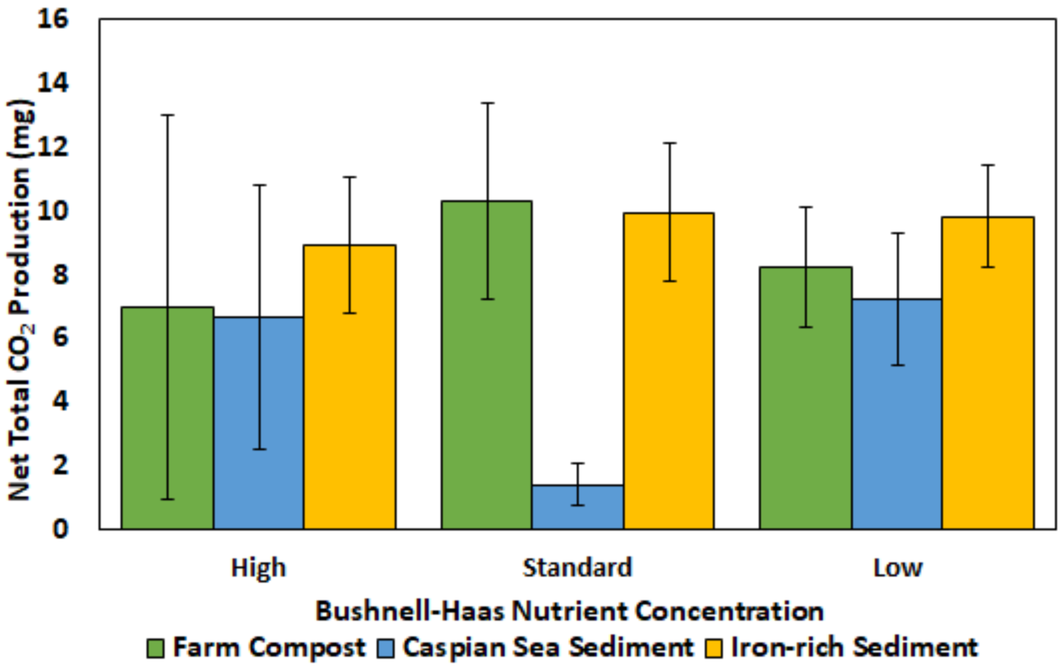

Figure S2. Total net CO_2 production after subtraction of the mean CO_2 production in mg from a control set of replicates containing no alkene input. Representing net CO_2 allows for more direct means of quantifying respiration attributable to alkene biodegradation.

PERMANOVA Comparison of 16S ASVs Across Inocula Type

Table S1. A summary of PERMANOVA results depicting R^2 values between the microbial communities across Caspian Sea sediment, iron-rich sediment, and farm compost inocula. The F-model values are shown in parentheses for each comparison. Bolded values indicate the p-value depicting the probability of >F for each comparison was <0.001.

	Caspian Sea Sediment	Iron-Rich Sediment	Farm Compost
Caspian Sea Sediment		**0.15052 (4.6069)**	**0.1588 (5.6635)**
Iron-Rich Sediment			**0.17444 (5.0711)**
Farm Compost			
